# Profiling Covid-19 patients with respect to level of severity: an integrated statistical approach

**DOI:** 10.1038/s41598-023-32089-3

**Published:** 2023-04-04

**Authors:** Federica Cugnata, Maria Giovanna Scarale, Rebecca De Lorenzo, Marco Simonini, Lorena Citterio, Patrizia Rovere Querini, Antonella Castagna, Clelia Di Serio, Chiara Lanzani

**Affiliations:** 1 University Centre of Statistics in the Biomedical Sciences, Vita-Salute San Raffaele University, Milan, Italy; 2 School of Medicine, Vita-Salute San Raffaele University, Milan, Italy; 3 Nephrology and Dialysis Unit, Genomics of Renal Diseases and Hypertension Unit, IRCCS San Raffaele Scientific Institute, Milan, Italy; 4 Division of Immunology, Transplantation and Infectious Diseases, IRCCS San Raffaele Scientific Institute, Milan, Italy; 5 Biomedical Faculty, Università Della Svizzera Italiana, Lugano, Switzerland

**Keywords:** Statistics, Nephrology, Infectious diseases

## Abstract

A full understanding of the characteristics of Covid-19 patients with a better chance of experiencing poor vital outcomes is critical for implementing accurate and precise treatments. In this paper, two different advanced data-driven statistical approaches along with standard statistical methods have been implemented to identify groups of patients most at-risk for death or severity of respiratory distress. First, the tree-based analysis allowed to identify profiles of patients with different risk of in-hospital death (by Survival Tree-ST analysis) and severity of respiratory distress (by Classification and Regression Tree-CART analysis), and to unravel the role on risk stratification of highly dependent covariates (i.e., demographic characteristics, admission values and comorbidities). The ST analysis identified as the most at-risk group for in-hospital death the patients with age > 65 years, creatinine $\ge$ 1.2 mg/dL, CRP $\ge$ 25 mg/L and anti-hypertensive treatment. Based on the CART analysis, the subgroups most at-risk of severity of respiratory distress were defined by patients with creatinine level $\ge$ 1.2 mg/dL. Furthermore, to investigate the multivariate dependence structure among the demographic characteristics, the admission values, the comorbidities and the severity of respiratory distress, the Bayesian Network analysis was applied. This analysis confirmed the influence of creatinine and CRP on the severity of respiratory distress.

## Introduction

It has been reported that patients with underlying disease are more likely to contract Coronavirus disease 19 (Covid-19) and become critically ill^1,2^. Older age, cardiovascular and kidney comorbidities, are among the most important risk factors influencing the virus–host interaction and the clinical outcome of Covid-19^3,4^. Understanding the relationship between comorbidities, therapy, and Covid-19 mortality is needed to efficiently guide clinical and public health interventions. Moreover, fully understanding the characteristics of Covid-19-related severity of respiratory distress is also necessary for an early identification and precise treatment^5^.

In this paper we aim at evaluating the effect of Covid-19 risk factors not only on in-hospital death but also on severity of respiratory distress. The novelty of our approach is to consider as severity outcome the variable SOFA (Sequential Organ Failure Assessment)^6^ defined on respiratory system according to 5 increasing levels of severity, a measure that accounts for the real individual Covid-19 evolution rather than for external factors. Indeed, very often Covid-19 severity outcome has been evaluated by means of Intensive Care Unit (ICU) binary outcome which may be misleading being affected both by the epidemic wave strength as well as by the ICU regional health policies. To this extent, our goal is to identify risk profiles of patients that might develop different severity of Covid-19 disease.

To pursue these aims, two different advanced statistical approaches were performed, along with standard statistical methods such as Cox and logistic regression analyses. The first data-driven approach, tree-based analysis, allowed to identify profiles of patients with different risks of in-hospital death and severity respiratory distress, and to unravel the role of highly dependent covariates on risk stratification. The second approach, the Bayesian Network analysis, was used to further explore the relationships among demographic characteristics, comorbidities, admission values and the severity of respiratory distress and to investigate the multivariate dependence structure.

## Materials and methods

### Study population

A single-center observational prospective cohort, the COVID-BioB study, was implemented at the IRCCS San Raffaele Scientific Institute in Milan, Italy.

Full description of patient management and clinical protocols were previously published^7^.

We included in the present study patients admitted to our hospitals from March 2 to April 25, 2020, with at least one plasma creatinine value measured during SARS-CoV-2 infection. A total of 392 consecutive patients were included in the present analysis. All patients aged 18 years or older, admitted to the IRCCS San Raffaele Scientific Institute with confirmed SARS-CoV-2 infection were consecutively enrolled in the COVID-BioB study. Diagnosis of SARS-CoV-2 infection was based on a positive real-time reverse-transcriptase polymerase chain reaction (RT-PCR) from a nasopharyngeal and/or throat swab or high clinical and radiological suspicion of Covid-19 pneumonia^7^.

The study was approved by the IRCCS San Raffaele Hospital Ethics Committee (protocol no. 34/int/2020) and was registered on ClinicalTrials.gov (NCT04318366).

For patients able to provide a signed informed consent at the time of hospital admission, written informed consent was obtained before data collection. Otherwise, patients consented as soon as they were able to sign. This study is reported in compliance with the STROBE statement^8^.

All methods were carried out in accordance with relevant guidelines and regulations.

### Data collection and definitions

Data were collected from medical charts review and entered in a dedicated COVID-BioB study electronic case record form (eCRF). The demographic characteristics, laboratory data and medications were extracted from electronic medical records. Before analysis data were cross-checked with medical charts and verified by data managers and clinicians for accuracy. The date of clinical observation start (baseline) was defined as the day of Emergency Department admission^7^.

Baseline serum creatinine was defined as the most recent available creatinine value in the previous 6 months in a stable clinical condition if available (21%) or the last value available previous discharge. For dead subjects, the minimum creatinine value during hospitalization after SARS-CoV-2 infection was selected. The acute kidney failure (AKI) was defined as a 50% increase in serum creatinine from baseline according to the KDIGO criteria^9^. Patients were defined as history of hypertension (HYP) if hypertension was reported in their medical history or if they were chronically treated at least one anti-hypertensive drug.

PaO_2_/FiO_2_ was used as an indicator of the severity of respiratory distress; it was recoded in 5 classes defined as by SOFA score^6,10,11^ (S1 Table).

### Statistical methods

Standard and advanced statistical techniques have been applied to identify the factors associated with an increased/decreased risk of in-hospital death and severity respiratory distress expressed as SOFA score. Along with standard Cox regression models, Survival Tree (ST) analysis has been implemented to identify risks factors associated with Covid-19 outcomes within a data-driven approach. The procedure allows to uncover profile of patients with different risk of in-hospital death and to disentangle the role of highly dependent covariates on risk stratification. The iterative ST algorithm selects the best predictors with the best thresholds aiming at identifying homogeneous subgroups of patients with similar survival outcome^12^. To account for overfitting, in the tree-building phase, a constraint was imposed by fixing the minimum number of observations in any terminal node at 20. This choice was motivated by the need to have enough observations in the nodes to properly carry out further analyses. Following ST analysis, the Kaplan–Meier method was used to estimate overall survival for each risk profile, and log-rank test has been applied to compare survival among groups of patients defined based on the risk profile^12^.

The variables used in the Cox regression models and ST analysis were the demographic characteristics and the admission values [i.e., age, sex, BMI, C-reactive protein (CRP) and creatinine], the comorbidities [i.e., coronary artery disease (CAD), diabetes, chronic obstructive pulmonary disease (COPD), malignancy (NPL), mean arterial blood pressure (MBP), AKI, HYP], the reduction of anti-hypertensive therapy after hospitalization and the severity of respiratory distress (expressed as PaO_2_/FiO_2_ and SOFA score).

Taking advantage of the ST analysis, the procedure was applied to also identify a cutoff value for the SOFA score to obtain a binary version of the score itself to be used in successive analyses.

To investigate the effects of comorbidities on severity of respiratory distress, univariate and multivariable logistic regression models were performed considering the same covariates as before (i.e., age, sex, BMI, CRP creatinine, CAD, diabetes, COPD, NPL, AKI, MBP, HYP and the reduction of anti-hypertensive therapy after hospitalization). Stepwise variable selection procedure was applied to identify a smaller set of relevant predictors.

In order to identify patients’ profiles with different risk of respiratory distress, based on the same covariates entered in the logistic regression model, another data-driven approach was used within the Classification and Regression Tree (CART) methodology which implements a form of binary recursive partitioning. A minimum number of observations in each terminal node was set at 20 to ensure sufficient observations in the nodes and to properly carry out further analyses.

Finally, also a Bayesian Network (BN) approach was used to explore the dependence structure among all variables included in the CART analysis. BNs are probabilistic graphical models showing the relationship among variables through a set of nodes, which are the variables, and arcs, which represent the relationships between them; the nodes that are not connected represent variables that are conditionally independent of each other. The directionality of the arcs is such that no directed cycles are included in the graph. Hence, BNs are considered as directed acyclic graphs (DAGs) and the parameters of the model represent the conditional probability distributions of each node for each combination of values of the preceding node(s)^13^.

The purpose of using BN in this research is to learn dependence structure directly from data, while excluding some directions among variables that are not feasible. The network structure has been estimated from data by a hill climbing algorithm with Akaike information criterion score functions.

This approach is essential to uncover complex interrelationships among variables and to gain a better insight into mechanisms involved in Covid-19 disease progression. While CART analysis reports best predictors and best splits allowing to classify patients based on their outcome, BN approach is here reported to integrate previous analyses highlighting how variables are related within the whole multivariate structure. Moreover, BNs allow for a better interpretation of results obtained from multivariate logistic and CART model, enabling to deeply investigate the role of some covariates on the outcome, thus evaluating how the conditioning on one or more variable impact and propagate on other variables in the network.

When we set a value of one or more variables in the network, we update the conditional probability distributions to reflect it. This updating is known as evidence propagation. Based on the estimated network, different possible scenarios can be examined by inserting and propagating new evidence on one or more variables throughout the network. Various diagnostic checks have been performed to investigate the effects of evidence on the distribution of a target variable using “what-if” sensitivity scenarios^14^.

Risks were reported as hazard ratios (HRs) or odds ratios (OR) along with their 95% Cis (Wald computation). A P value < 0.05 was considered significant. All analyses were performed using R statistical software (version 4.0.4; https://cran.r-project.org/index.html). The R package rpart was used to implement ST and CART analyses. The procedure applies the LeBlanc and Crowley splitting rule. The R packages bnlearn^15^ and gRain^16^ were used to learn the network and perform the inference required to calculate the conditional probabilities.

## Results

Demographic and clinical characteristics of 392 patients included in the study are shown in Table 1. In our cohort, during in-hospital stay (median 10 days, IQR 15–6 days) 95 deaths occurred. The mean age of patients was 66 years, 75% were male and 37% have reduced anti-hypertensive therapy during the hospitalization (Table 1).

**Table 1:** Demographic and clinical characteristics of study patients with SARS-CoV-2 infection.

	N	
Anthropometric measurements		
Males (n, %)	392	293 (74.7)
Age at recruitment (yrs)	392	65.65 ± 13.1
Age at recruitment > 65 years (n, %)	392	207 (52.8)
BMI (kg/m^2^)	309	27.05 ± 4.7
Underweight and normal BMI (n, %)		114 (36.9)
Overweight BMI (n, %)	309	129 (41.7)
Obese BMI (n, %)		66 (21.4)
Clinical history		
CAD (n, %)	381	103 (27.0)
HYP (n, %)	381	202 (53.0)
Diabetes (n, %)	381	68 (17.8)
COPD (n, %)	386	28 (7.2)
NPL (n, %)	381	54 (14.2)
Admission values		
MBP (mmHg)	373	91.08 ± 11.93
CRP (mg/L)	270	79.75 (31.7–139.9)
Creatinine (mg/dL)	392	1.01 (0.82–1.23)
SatO_2_ (%)	340	94 (91–96)
PaO_2_/FiO_2_ (mmHg)	304	290.5 (223.2, 328.6)
AKI (n, %)	380	21 (5.5)
In-hospital values		
Reduced anti-hypertensive therapy	274	101 (36.9)
Outcomes		
In-hospital mortality (n, %)	392	95 (24.2)
SOFA = 0 (≥ 400 mmHg) (n, %)		7 (2.3)
SOFA = 1 (< 400 mmHg) (n, %)		128 (42.1)
SOFA = 2 (< 300 mmHg) (n, %)	304	107 (35.2)
SOFA = 3 (< 200 mmHg and MV) (n, %)		35 (11.5)
SOFA = 4 (< 100 mmHg and MV) (n, %)		27 (8.9)
SOFA > 2 (n, %)	304	62 (20.4)

Continuous variables were reported as mean ± SD whereas categorical variables as total frequencies and percentages. Skewed variables are presented as median (interquartile range).

*CAD* coronary artery disease, *HYP* history of hypertension or use of at least one anti-hypertensive drug*, COPD* chronic obstructive pulmonary disease, *NPL* malignancy, *CRP* C-reactive protein, *MBP* mean arterial blood pressure, *SatO*_*2*_ peripheral arterial oxygen saturation, *AKI* acute kidney failure, *MV*, mechanical ventilation.

SOFA score was evaluated on *PaO*_*2*_*/FiO*_*2*_ values (where *PaO*_*2*_ partial pressure of arterial oxygen and *FiO*_*2*_ fraction of inspired oxygen).

### Risk of in-hospital death

Univariate and multivariable Cox regression analyses were reported in Supplementary Materials (S2 Table and S3 Table). The multivariable model fitted using stepwise selection show that older age (> 65 years), HYP, presence of COPD, higher values of creatinine and SOFA score > 2 were associated with a higher risk of death.

**Figure 1:**
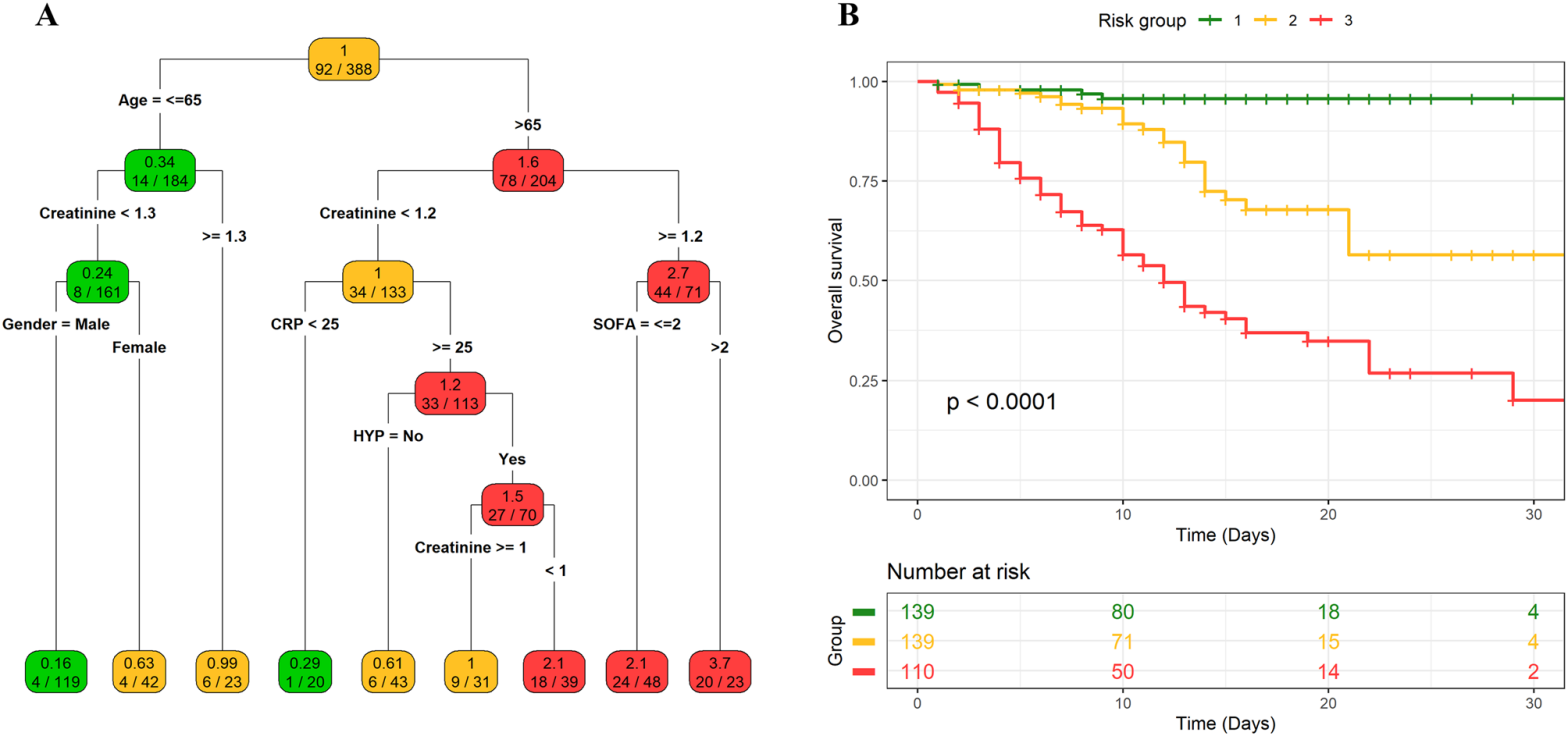
ST analysis and Kaplan–Meier curves and log-rank test. (**A**) ST analysis and (**B**) Kaplan–Meier curves and log-rank test for the three risk groups obtained from the ST analysis based on their HR computed in the final nodes. The low-risk group includes those patients falling in final nodes with an HR lower than 0.5 (n = 139, 36%), patients in the medium-risk group are those with an HR between 0.5 and 1 (n = 139, 36%), and patients in the high-risk groups are those with an HR higher than 1 (n = 110, 28%). The P value associated with the log-rank test is also displayed.

To uncover natural and homogeneous groups of subjects with similar survival outcome, we applied ST analysis that considered all baseline characteristics and comorbidities at admission and, for reduced anti-hypertensive therapy, the change during hospitalization. The ST analysis selected age, creatinine, SOFA score (> 2), sex, CRP, and HYP (Fig. 1A).

The main discriminant was age, with a favourable outcome in patients ≤ 65 years old. The second discriminant was creatinine. In younger patients, creatinine levels > 1.2 mg/dL was associated with unfavourable outcome regardless of gender. In patients with > 65 years old only if simultaneously creatinine levels were < 1.2 mg/dL and CRP < 25 mg/L the individual risk was equal to 0.29. Conversely, when CRP levels were higher than 25 mg/L and patients is a hypertensive individual, the risk increased to 2.1. On the other hand, the higher risk of in-hospital death was observed in patients with creatinine levels ≥ 1.2 mg/dL (HR = 2.7): as expected a higher risk was observed when patients underwent mechanical ventilation (i.e., SOFA score > 2) reaching an HR of 3.7.

Thus, the ST analysis identified the different patterns associated with different clinical outcomes. The concordance index of the Cox model considering the nine groups identified by the final nodes is equal to 0.778. Based on the HR in the final nodes, leaves were grouped to obtain three risk stratification categories (Fig. 1B): HR lower than 0.5 (n = 139, 36%), HR between 0.5 and 1 (n = 139, 36%), and HR higher than 1 (n = 110, 28%), showing a marked difference in terms of survival (P < 0.001; Fig. 1B).

### Risk of severity of respiratory distress

ST analysis for SOFA and in-hospital death identified a cutoff equal to 2 (i.e., PaO_2_/FiO_2_ < 300 mmHg), corresponding to the need of mechanical ventilation (at admission 20% of patients had SOFA score > 2). Then SOFA score was dichotomized considering value of 2.

To investigate the role of comorbidities in increasing risk of mechanical ventilation, univariate and multivariable logistic regressions were performed. Age > 65 years, history of hypertension and higher levels of CRP and creatinine were associated with an increased risk of mechanical ventilation (S4 Table). After stepwise selection, the fully adjusted model comprised only age and CRP levels, both of which were positively associated with the risk of mechanical ventilation (Table 2).

**Table 2:** Significant associations resulting from stepwise selection on multivariable logistic regression (n = 216, events = 37).

	OR	95% CI		p-value
		LB	UB	
Age at recruitment > 65 yrs (yes vs. no)	2.50	1.11	5.62	2.67 × 10^–2^
CRP (per 10 mg/L)	1.09	1.04	1.14	2.81 × 10^–4^

*CRP* C-reactive protein.

Outcome: SOFA > 2; covariates: age, sex, BMI, CRP, creatinine, CAD, diabetes, COPD, NPL, MBP, AKI, HYP and the reduction of anti-hypertensive therapy after hospitalization.

**Figure 2:**
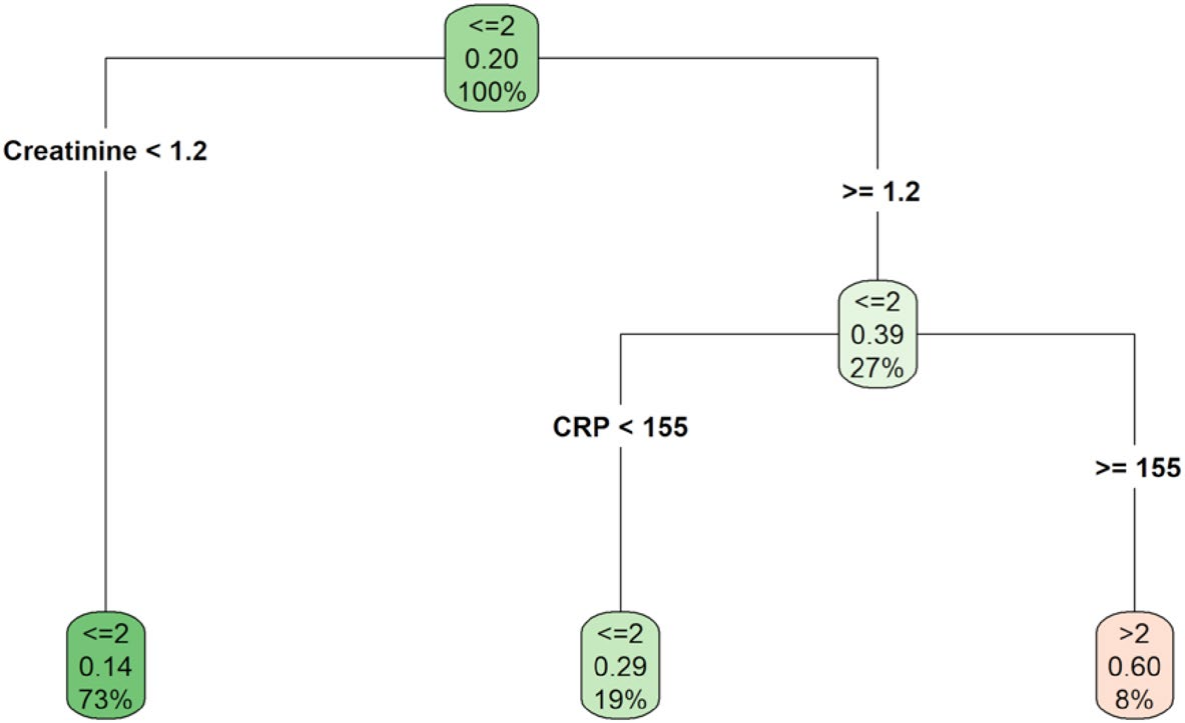
Classification regression tree. Outcome: SOFA > 2; covariates: age, sex, BMI, CRP, creatinine, CAD, diabetes, COPD, NPL, MBP, AKI, HYP and the reduction of anti-hypertensive therapy during hospitalization.

In order to define the patient profile at higher risk of mechanical ventilation, CART analysis was carried out. In this case, a creatinine level below 1.2 mg/dL defined subgroups of patients with the lowest risk of adverse outcomes (probability of being SOFA > 2 = 0.14) (Fig. 2).

Among the patients with creatinine ≥ 1.2 mg/dL, the individual risk was 0.29 in patients with CRP levels lower than 155 mg/L and 0.60 in those with CRP levels greater than 155 mg/L. The accuracy rate of the estimated CART is equal to 81.25%.

**Figure 3:**
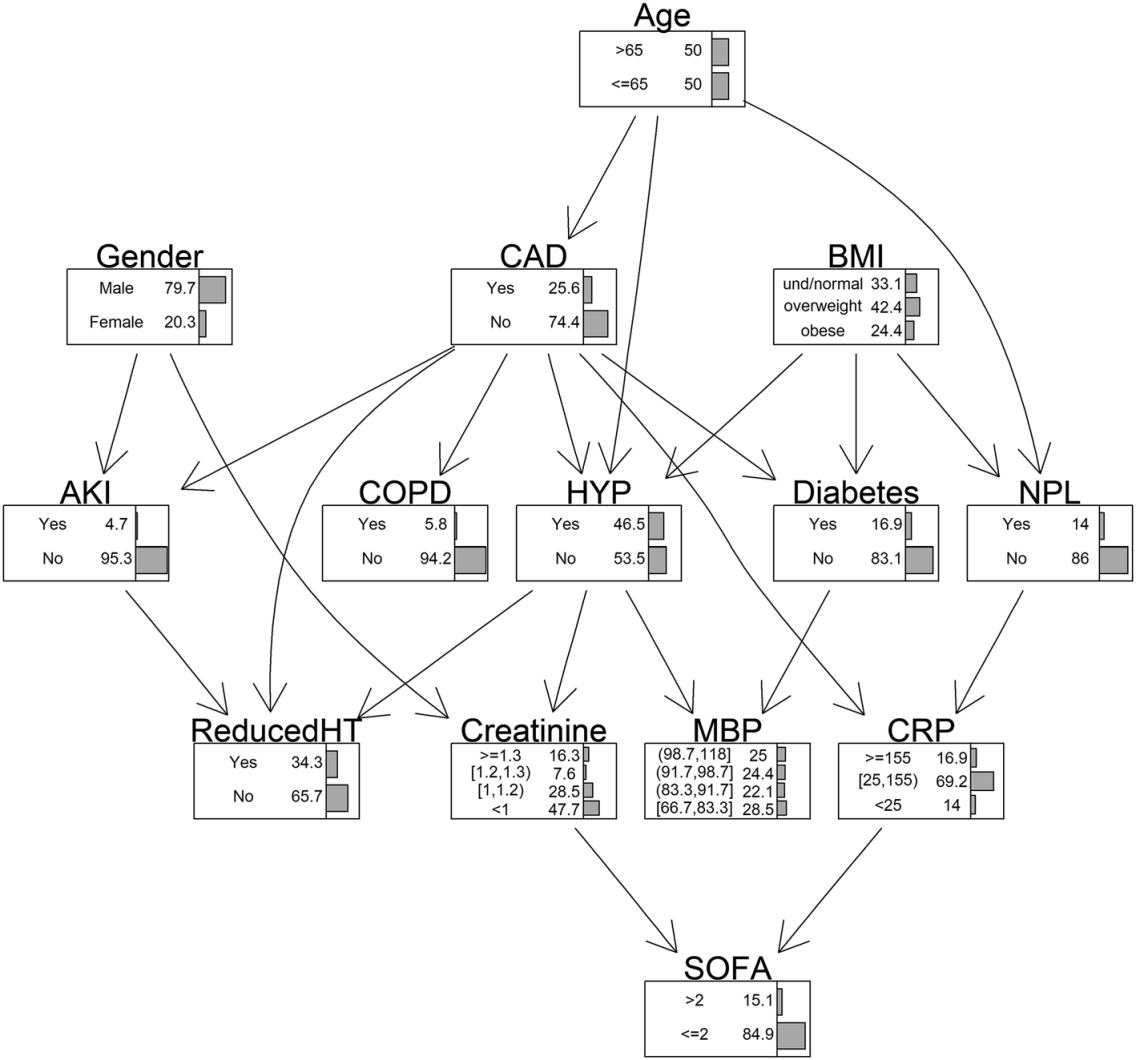
Bayesian Network Model for SOFA with marginal probability tables expressed in percentage. Outcome: SOFA > 2; covariates: age, sex, BMI, CRP, creatinine, CAD, diabetes, COPD, NPL, MBP, AKI, HYP and the reduction of anti-hypertensive therapy after hospitalization.

To further explore the relationships among demographic characteristics, comorbidities, admission values and the severity of respiratory distress, BNs were applied to investigate their multivariate dependence structure. The resulting network is shown in Fig. 3 where the variables are represented in the network nodes together with its corresponding marginal probability table, expressed as percentage.

Moving through the network from one node to another it is possible to evaluate which variables influence directly or indirectly the target ones. For example, SOFA (the severity of respiratory distress) is directly affected by creatinine and CRP, and it is indirectly influenced by many other variables, such as gender and HYP which showed a direct effect on creatinine and CAD and NPL which showed a direct effect on CRP. Identifying which features have a direct or indirect influence on the target variable is of particular importance to better understand how risks factors affect Covid-19 outcomes and to plan and to develop improvement strategies.

**Figure 4:**
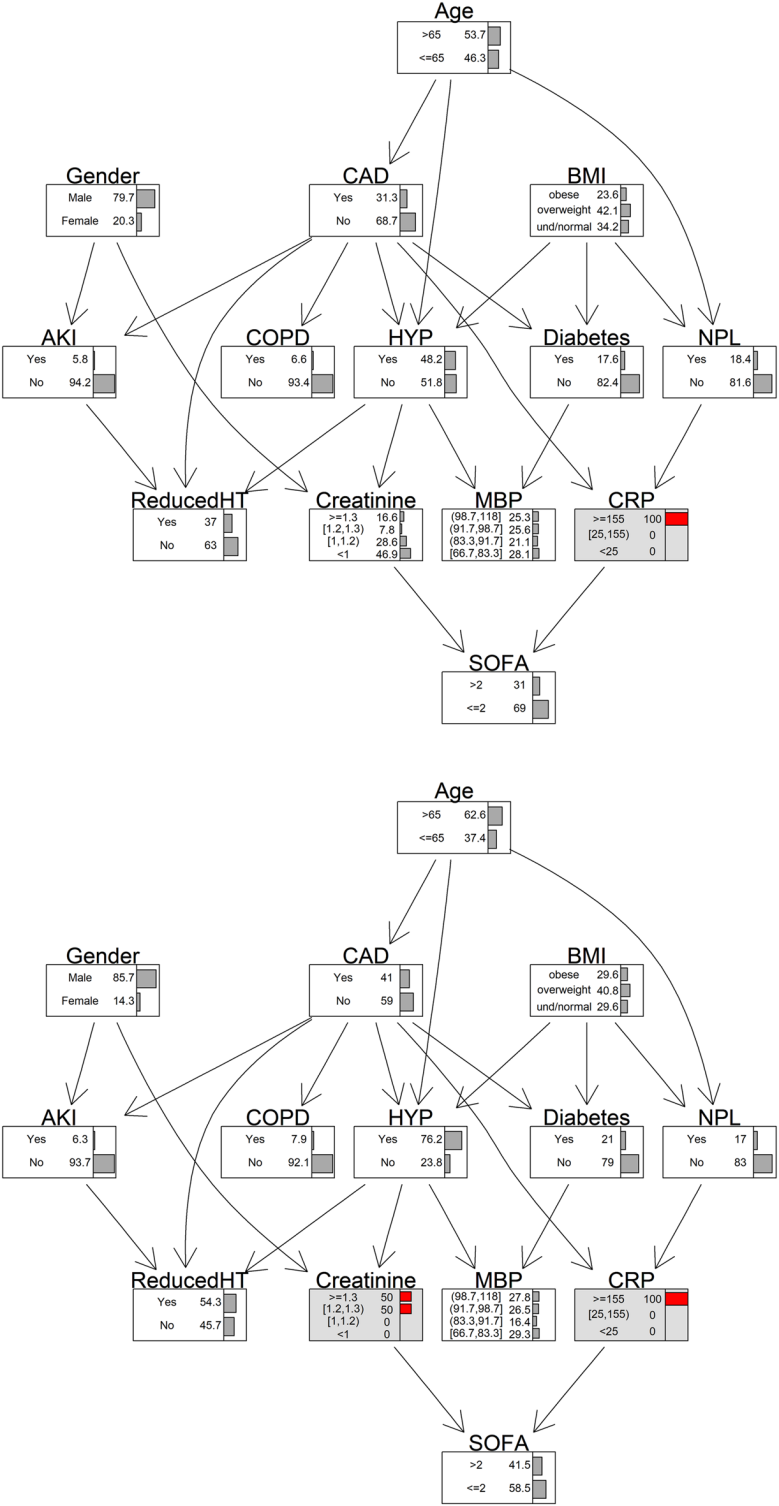
Hypothetical scenarios. Conditioned on CRP ≥ 155 mg/L and conditioned on CRP ≥ 155 mg/L and creatinine ≥ 1.2 mg/dL.

Once the model has been estimated, a key feature of the BN approach is that it provides the opportunity to assess alternative hypothetical scenarios. From the clinical point of view, it is interesting to evaluate which factors produce an increasing effect on the risk of severity of respiratory distress. By fixing CRP at the maximum level (≥ 155 mg/L), the probability to observe SOFA > 2 increases from 0.16 to 0.31 (Fig. 4) while by fixing both CRP at the maximum level (≥ 155 mg/L) and creatinine ≥ 1.2 mg/dL the probability to observe SOFA > 2 increases to 0.41 (Fig. 4). This finding emphasizes the role of BNs not only in measuring the dependence structure but also in highlighting the sign and strength of the interaction among risk factors by means of conditional probabilities.

## Discussion

In Covid-19 patients one major hurdle have been how to define patients’ severity. Indeed, many classifications (like binary classification for ICU admission, or level of respiratory impairments) suffer from being affected by specific emergency in hospitalization and difficulty. The main goal of this paper was to profile Covid-19 patients with respect to disease severity with an integrated statistical approach relying on machine learning and Bayesian networks to investigate general dependence structure of the data.

This allowed us to stratify patients with respect to their risk of experiencing worst outcome (i.e., in-hospital death and severity of respiratory distress). Results obtained with different approaches were consistent in confirming not only the well-known strong association between older age, CRP, SOFA score and death in Covid-19^17^ but also in founding a lower creatinine cutoff (i.e., 1.2 mg/dL) than the currently found in literature^18,19^. This suggests a Covid-19 specific threshold in creatinine which is lower than the common threshold of severe chronic kidney disease, thus spreading light on a possible new crucial role of creatinine as early predictor of severe Covid-19 disease. The selected threshold equal to 1.2 mg/dL coincides with the threshold of attention for nephrologists for incoming kidney impairment (typically a creatinine level greater than 1.2 mg/dL for women and greater than 1.4 mg/dL for men, corresponding to approximately eGFR less than 60 ml/min/1.73 m^2^)^9^.

Another important finding that emerged from these analyzes is the role of the SOFA score, assessed both as exposure and as outcome. It is well known that SOFA score is associated with mortality in Covid-19^20^ and when it was considered as prognostic factor in ST, the cut-off value (i.e., > 2) identified in literature was confirmed^21^. On the other hand, when SOFA score was evaluated as outcome, creatinine level less than 1.2 mg/dL identified the group of patients with low risk of severe respiratory distress. These results were also confirmed by BN analyses.

The multilayer definition of risk defined by the ST and CART analyses represents a framework to implement the principles of precision medicine in the management of the Covid-19 pandemic and can be used to identify patients at risk in the context of clinical trials or public health interventions. Prognostic factors (stand-alone or combined in risk assessment models) may guide the stratification of patients with SARS-CoV-2 infectious disease based on their risk of severe respiratory distress or death. This risk stratification may subsequently guide optimized management and resource utilization strategies in the care of these patients.

## Supplementary Information

Supplementary Tables.

## Data Availability

The datasets analyzed during the current study available from the corresponding author on reasonable request.
