# Supplementary material for: Profiling Covid-19 patients with respect to level of severity: an integrated statistical approach
Source: Sci Rep. 2023 Apr 4;13:5498. doi: 10.1038/s41598-023-32089-3 (PMC10071456; doi:10.1038/s41598-023-32089-3)
Supplement: Supplementary file 1 — Supplementary Tables. [file 41598_2023_32089_MOESM1_ESM.docx]

**Supporting Information**

**S1 Table. Respiratory system**

| **PaO_2_/FiO_2_ [mmHg (kPa)]** | **SOFA score** |
| --- | --- |
| ≥ 400 (53.3) | 0 |
| < 400 (53.3) | 1 |
| < 300 (40) | 2 |
| < 200 (26.7) **and** mechanically ventilated | 3 |
| < 100 (13.3) **and** mechanically ventilated | 4 |

**S2 Table. Univariate associations with in-hospital death in patients with SARS-CoV-2 infection**

| **Covariate** | **n** | **events** | **HR** | **95% CI** | | **p-value** |
| --- | --- | --- | --- | --- | --- | --- |
|  |  |  |  | **LB** | **UB** |  |
| Males vs. females | 392 | 95 | 0.96 | 0.59 | 1.58 | 8.79x10^-1^ |
| Age at recruitment > 65 yrs (yes vs. no) | 392 | 95 | 5.17 | 2.93 | 9.12 | 1.38x10^-8^ |
| BMI (per 1 unit) | 309 | 53 | 0.94 | 0.88 | 1.01 | 8.38x10^-2^ |
| MBP (per 5 mmHg) | 373 | 83 | 0.88 | 0.8 | 0.97 | 9.50x10^-3^ |
| HYP (yes vs. no) | 381 | 89 | 4.30 | 2.50 | 7.40 | 1.27x10^-7^ |
| Reduced hypertensive therapy (yes vs. no) | 274 | 52 | 3.35 | 1.91 | 5.88 | 2.50x10^-5^ |
| CAD (yes vs. no) | 381 | 89 | 3.11 | 2.05 | 4.73 | 1.01x10^-7^ |
| Diabetes disease (yes vs. no) | 381 | 89 | 1.77 | 1.11 | 2.83 | 1.62x10^-2^ |
| COPD (yes vs. no) | 386 | 90 | 3.95 | 2.35 | 6.63 | 2.07x10^-7^ |
| NPL (yes vs. no) | 381 | 89 | 1.97 | 1.2 | 3.23 | 7.57x10^-3^ |
| AKI (yes vs. no) | 380 | 86 | 2.3 | 1.22 | 4.33 | 1.02x10^-2^ |
| Creatinine (per 1 mg/dL) | 392 | 95 | 1.47 | 1.29 | 1.67 | 6.56x10^-9^ |
| CRP (per 10 mg/L) | 270 | 52 | 1.04 | 1.01 | 1.07 | 1.40x10^-2^ |
| PaO_2_/FiO_2_ (per 5 unit) | 304 | 70 | 0.97 | 0.96 | 0.99 | 1.24x10^-5^ |
| SOFA >2 (yes vs. no) | 304 | 70 | 2.97 | 1.85 | 4.77 | 7.17x10^-6^ |

*CAD* coronary artery disease, *COPD* chronic obstructive pulmonary disease, *NPL* malignancy, *MBP* mean arterial blood pressure, *HYP* history of hypertension or use of at least one anti-hypertensive drug, *AKI* acute kidney failure, *CRP* C-reactive protein, *SatO_2_* Peripheral arterial oxygen saturation, *SOFA* score was evaluated on *PaO_2_/FiO_2_* values (where *PaO_2_* partial pressure of arterial oxygen and *FiO_2_* fraction of inspired oxygen).

**S3 Table. Multivariable associations with in-hospital death in patients with SARS-CoV-2 infection (after stepwise selection) (n=299, events=69)**

| **Covariate** | **HR** | **95% CI** | | **p-value** |
| --- | --- | --- | --- | --- |
|  |  | **LB** | **UB** |  |
| Age at recruitment > 65 yrs (yes vs. no) | 2.50 | 1.29 | 4.85 | 6.80x10^-3^ |
| HYP (yes vs. no) | 2.10 | 1.13 | 3.93 | 1.95x10^-2^ |
| COPD (yes vs. no) | 3.02 | 1.67 | 5.47 | 2.50x10^-4^ |
| Creatinine (per 1 mg/dL) | 1.29 | 1.07 | 1.56 | 6.49x10^-3^ |
| SOFA > 2 (yes vs. no) | 2.56 | 1.55 | 4.22 | 2.40x10^-4^ |

*COPD* chronic obstructive pulmonary disease, *HYP* history of hypertension or use of at least one anti-hypertensive drug. *SOFA* score was evaluated on PaO_2_/FiO_2_ values.

Outcome: in-hospital death; covariates: age, sex, BMI, CRP, creatinine, CAD, diabetes, COPD, NPL, MBP, AKI, HYP and the reduction of anti-hypertensive therapy after hospitalization, SOFA.

**S4 Table. Univariate associations with mechanical ventilation (outcome: SOFA>2) in patients with SARS-CoV-2 infection**

| **Covariate** | **n** | **events** | **OR** | **95% CI** | | **p-value** |
| --- | --- | --- | --- | --- | --- | --- |
|  |  |  |  | **LB** | **UB** |  |
| Males vs. females | 304 | 62 | 1.53 | 0.77 | 3.05 | 2.28x10^-1^ |
| Age at recruitment > 65 yrs (yes vs. no) | 304 | 62 | 2.44 | 1.35 | 4.39 | 3.05x10^-3^ |
| BMI (per 1 unit) | 245 | 47 | 1.04 | 0.97 | 1.11 | 2.68x10^-1^ |
| MBP (per 5 mmHg) | 301 | 59 | 0.98 | 0.87 | 1.11 | 7.67x10^-1^ |
| HYP (yes vs. no) | 299 | 62 | 2.03 | 1.14 | 3.62 | 1.67x10^-2^ |
| Reduced hypertensive therapy (yes vs. no) | 218 | 39 | 1.62 | 0.8 | 3.26 | 1.79x10^-1^ |
| CAD (yes vs. no) | 299 | 62 | 1.43 | 0.77 | 2.65 | 2.58x10^-1^ |
| Diabetes disease (yes vs. no) | 299 | 62 | 1.18 | 0.58 | 2.42 | 6.47x10^-1^ |
| COPD (yes vs. no) | 304 | 62 | 1.62 | 0.6 | 4.37 | 3.39x10^-1^ |
| NPL (yes vs. no) | 299 | 62 | 1.91 | 0.88 | 4.16 | 1.01x10^-1^ |
| AKI (yes vs. no) | 295 | 58 | 0.67 | 0.15 | 3.08 | 6.06x10^-1^ |
| Creatinine (per 1 mg/dL) | 304 | 62 | 1.35 | 1 | 1.8 | 4.79x10^-2^ |
| CRP (per 10 mg/L) | 216 | 37 | 1.09 | 1.05 | 1.14 | 7.84x10^-5^ |

*CAD* coronary artery disease, *COPD* chronic obstructive pulmonary disease, *NPL* malignancy, *MBP* mean arterial blood pressure, *HYP* history of hypertension or use of at least one anti-hypertensive drug, *AKI* acute kidney failure, *CRP* C-reactive protein.
